# The Heterogeneity, Origins, and Impact of Migratory iILC2 Cells in Anti-helminth Immunity

**DOI:** 10.3389/fimmu.2020.01594

**Published:** 2020-07-23

**Authors:** Mindy M. Miller, R. Lee Reinhardt

**Affiliations:** ^1^Department of Biomedical Research, National Jewish Health, Denver, CO, United States; ^2^Department of Immunology and Microbiology, University of Colorado-Anschutz Medical, Aurora, CO, United States

**Keywords:** iILC2, nILC2, ILC2, helminth, *Nippostrongylus brasiliensis*, IL-4, IL-13, type-2 immunity

## Abstract

Soil-transmitted helminths represent a major global health burden with infections and infection-related comorbidities causing significant reductions in the quality of life for individuals living in endemic areas. Repeated infections and chronic colonization by these large extracellular worms in mammals led to the evolution of type-2 immunity characterized by the production of the type-2 cytokines interleukin (IL)-4, IL-5, and IL-13. Although a number of adaptive and innate immune cells produce type-2 cytokines, a key cellular source in the context of helminth infection is group 2 innate lymphoid cells (ILC2s). ILC2s promote mucosal barrier homeostasis, integrity, and repair by rapidly responding to epithelial cues in mucosal tissues. Though tissue-resident ILC2s (nILC2s) have been studied in detail over the last decade, considerably less is known with regard to a subset of inflammatory ILC2s (iILC2s) that migrate to the lungs of mice early after *Nippostrongylus brasiliensis* infection and are potent early producers of type-2 cytokines. This review will discuss the relationship and differences between nILC2s and iILC2s that establish their unique roles in anti-helminth immunity. We have placed particular emphasis on studies investigating iILC2 origin, function, and their potential long-term contribution to tissue-resident ILC2 reservoirs in settings of helminth infection.

## Introduction

### Global Health Burden of Soil-Transmitted Helminths

Soil-transmitted helminths include roundworms (*Ascaris lumbricoides*), whipworms (*Trichuris trichiura*) and hookworms (*Necator americanus; Ancylostoma duodenale*) together accounting for infections in at least 1.4 billion people currently, with half of the world's population remaining at risk (1–3). Hookworm transmission is initiated when fertilized eggs are excreted in the feces of infected hosts and hatch in the environment to release infectious larvae. When bare skin is exposed to fecal-contaminated soil, hookworm larvae penetrate the skin, enter the circulation of the host, and migrate to the lungs. Here they enter the parenchyma via pulmonary alveolar capillaries, differentiate and move into pulmonary airspaces, ascend the pharynx, and are swallowed. Larvae eventually take residence in the lumen of the small intestine where they mature into adults and lay eggs. The exception to this lifecycle is the hookworm *Anclostoma duodenale*, which can be transmitted through the direct ingestion of larvae instead of epithelial penetration.

While mortality due to STH is concerning, the massive clinical burden associated with helminths manifests from the comorbidities related to anemia, abdominal pain, diarrhea, dehydration, and physical and cognitive growth retardation (4–6). It is estimated that combined STH infection contributes to 5–14 million disability-adjusted life years (DALYs) which disproportionately affects low-income economies already stressed with unmet health care needs (3, 7, 8). However, even this is likely an underestimation of the overall burden associated with STH infections (9). One DALY equates to the loss of 1 year of “healthy” life over the lifetime of an individual. Although reports of infection rates and DALYs vary, hookworms alone are estimated to infect 400 million individuals and are responsible for 3.2 million DALYs (1, 7). This is concerning in particular for school-aged children as hookworm infection has long been associated with reduced cognitive function and academic performance (10–13). Moreover, mothers infected with helminths even a single time during pregnancy may give birth to infants with impaired cognitive and gross motor function (14). Unfortunately, this early-life impairment could lead individuals on a trajectory toward lower productivity and earning capacity when reaching adulthood (15). These comorbidities are compounded by nutrition deficits associated with high helminth burdens. Chronic and repeated helminth colonization has been described as the “world's most important nutrition problem” (16).

Preventative measures have proven highly successful in limiting helminth infections among industrialized nations. This may be best demonstrated by the reduction in the incidence in *Necator americanus* infections within the southern United States following the Rockefeller Sanitary Commissions' influence in implementing better hygiene practices and access to anthelmintics starting in the early 1900s (17). As a result of these practices, hookworm infection in the United States was effectively eliminated (18). A similar effect was observed during the industrialization of Japan (18). However, in developing nations and more rural regions of the world where basic sanitary needs—such as running water and sewage treatment plants—are lacking and where access to drugs is limited, such preventative measures have been less successful (3, 19, 20). This is despite the continued goal of the World Health Organization to eliminate STH infections as a public health concern (21). The majority of the WHO's approach has been based on mass chemotherapeutic approaches that would provide regular treatment to 75% of school age children living in regions endemic to helminth infections. In support of such an approach, several pharmaceutical agents exist to eliminate STH infections, the most common being benzimidazoles which kill the parasite through preventing microtubule polymerization. Although anthelmintic drugs have proven effective at eliminating current infections, they are not preventative and must be re-administered for each subsequent infection (6). As such, repeated treatments have been deemed ineffective in many of the low-socioeconomic communities due to costs associated with frequent clinic visits and the logistics of providing routine access to drugs within key endemic populations. For example, 39 countries do not yet meet the 75% treatment goal, and when preschool-aged children are included in the target demographic, <50% of children are receiving the expected chemotherapy regimen (3, 22). Even among children receiving treatment, the high rates of reinfection and inconsistent access to drugs has made the goal of helminth eradication by this approach difficult to achieve (5, 23–25). Furthermore, there is increasing concern that sporadic chemotherapeutic interventions will increase the incidence of drug-resistance, particularly in nematodes (20, 26, 27). Additional concern lies in side effects associated with these drugs. Even though most anthelmintics are well-tolerated, side effects include gastrointestinal discomfort and the high doses required to treat echinococcal liver cysts have been associated with hair loss, bone marrow suppression, and hepatic injury (28). Thus, while advances in anthelmintic drugs are likely to continue to make a positive impact on the global burden of helminth-related morbidity, there is a continued need for additional pharmaceutical agents and/or vaccines tailored toward the safe prevention/elimination of infections. Novel approaches to limit infection and worm burden would benefit from a more complete understanding of the immune response to helminths.

### Type-2 Inflammation and Anti-helminth Immunity

The majority of animal studies assessing immunity to soil-transmitted helminths utilize either the murine hookworm *Nippostrongylus brasiliensis*, which mimics the lifecycle of the human pathogen *Necator americanus*, or the fecal-oral, intestinal roundworm model of *Heligomosomoides polygyrus*. Whether assessing the lung or the intestine in these models, a characteristic wound healing or tissue repair response is observed and is referred to as type-2 immunity (29). Although type-2 immune responses are often observed in the context of allergic asthma, this form of immunity likely evolved to protect the host from comorbidities associated with chronic or repeated helminth exposure and is ideally suited to promoting parasite clearance and tissue repair (30–32). While the relationship between anti-helminth immunity and allergic disease is complex, evidence suggests that despite invoking similar type-2 inflammatory processes, helminth infection does not always exacerbate allergic inflammation (33). In fact, recent literature shows that there is likely an early-life window that can be exploited to influence an individual's susceptibility to chronic diseases including asthma in later life (34). Such helminth-mediated suppression of allergic immunity would support the observation that individuals living in rural areas endemic to helminth infection develop allergic disease in a smaller percentage of the population than do individuals living in more industrialized communities that are devoid of parasitic helminths (35–38). Empirical studies also support this conclusion (39–42). Mice infected with various parasitic helminths, including *Heligmosomoides polygyrus, Nippostrongylys brasiliensis*, and *Litomosoides sigmodontis*, show reduced allergic lung disease when sensitized and challenged with allergens. Together, the evidence suggests that mammals evolved suppressive mechanisms that work in concert with type-2 inflammation to tolerate and/or clear helminth infection. These mechanisms are likely advantageous to individuals by limiting the damage induced by repeated worm infection and colonization. As such, allergy and asthma may be more recent manifestations of type-2 inflammation in hosts that lack the natural exposure to these parasitic worms (43, 44). In this case, the “poised” type-2 immunity designed to tolerate helminths now responds inappropriately to innocuous allergens. This represents an important variation of the hygiene hypothesis (45–47).

Type-2 immunity is orchestrated through the production of the type-2 cytokines interleukin (IL)-4, IL-5, and IL-13 (48). These three cytokines have both unique and redundant roles in anti-helminth immunity. IL-4 produced by follicular helper T cells in the context of helminth infection is critical for the production of immunoglobulin (Ig) E and high-affinity IgG1 (49–51). Although the crosslinking of IgE receptors on basophils and mast cells appears limited in primary *N. brasiliensis* infection, IgE-mediated activation of basophils is likely more extensive during secondary helminth responses (52, 53). In addition, IL-4 from basophils and eosinophils also promotes type-2 inflammation at the site of infection/colonization (54–56). Although IL-13 likely plays a role within pathogenic IgE response to allergens, it does not appear to affect the production of IgE during acute helminth infection (57, 58). Instead, IL-13 primarily acts on the epithelium at mucosal barriers. Specifically, IL-13 can enhance goblet and tuft cell hyperplasia, increase mucus production, accelerate epithelial cell turnover, and aid in smooth muscle contractility in settings of type-2 inflammation (57, 59–62). IL-13 appears to be dominant to IL-4 in these processes primarily due to two factors. First, while the IL-4 receptor found on goblet cells and smooth muscle cells can bind both IL-4 and IL13, it has higher affinity for IL-13 (63, 64). Second, while IL-4 is the dominant cytokine produced in lymphoid tissues during helminth infection, IL-13 appears to be more restricted to immune cells residing in mucosal tissues (50, 57). This increases the availability of IL-13 to modulate goblet cell hyperplasia and smooth muscle contractility. Furthermore, type-2 cytokines can induce the production of downstream epithelial cytokines that are equally important in pathogen clearance. For example. IL-4/IL-13-induced goblet cells produce the cytokine RELM-beta which can directly impair helminth fecundity and survival (65, 66). Unlike IL-4 and IL-13—which can compensate to promote characteristic type-2 hallmarks (67, 68)—IL-5 appears to have a more specific role in type-2 responses confined primarily to the mobilization of eosinophils from the bone marrow to enhance wound healing and further increase IL-4 levels as they are the most prevalent IL-4-competent population at the peak of the *N. brasiliensis* response (56). Moreover, eosinophils likely play a role in direct killing of infectious larvae, particularly after reinfection (69).

The biologic importance of IL-4, IL-5, and IL-13 in anti-helminth immunity is best evidenced by changes in helminth worm clearance among cytokine-deficient mice. In support of IL-13 being the dominant type-2 cytokine in promotion of worm expulsion, IL-13-deficient mice displayed delayed helminth clearance compared to IL-4-deficient animals (70, 71). However, mice lacking both IL-4 and IL-13 exhibited a greater deficit in worm clearance than that observed in IL-13 single knockouts, indicating synergy of these type-2 cytokines in the context of *N. brasiliensis* infection (72). This result was phenocopied in mice where IL-13-producing cells were deleted upon diphtheria toxin administration (57). Importantly, IL-4/IL-5/IL-13-triple knockout mice show exceptionally delayed *N. brasiliensis* clearance (73). Together these cytokines perform three key functions important to anti-helminth immunity. First, they promote the mobilization of innate and adaptive type-2 immune cells to sites of helminth infection and mucosal barrier damage. Second, they work synergistically and independently to induce the weep (mucus production) and sweep (smooth muscle contractility) response to mechanically expel the worms. Lastly, these cytokines are involved in the repair of damaged epithelium that results during helminth migration and colonization. Although IL-4, IL-5, and IL-13 are responsible for the majority of type-2 immune hallmarks observed during helminth infection, IL-9 has been described to have additional effects on worm clearance (73, 74).

**Figure 1:**
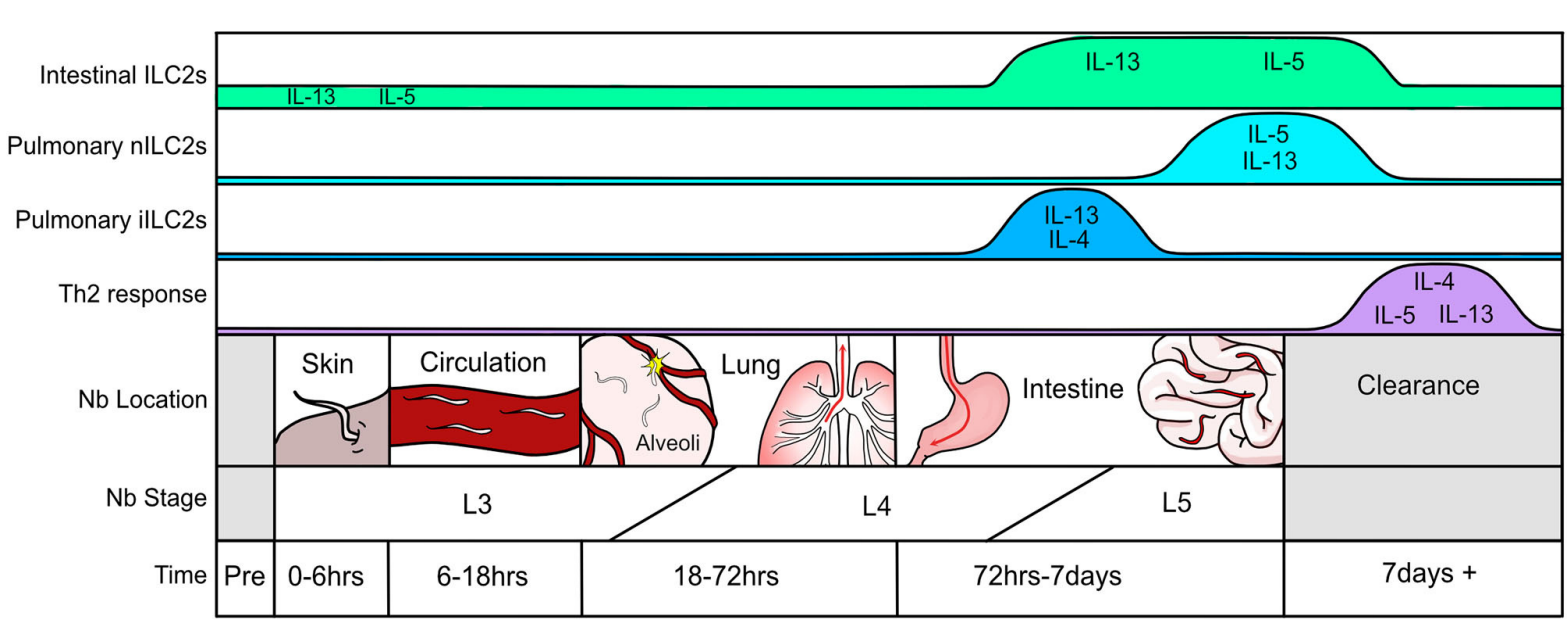
Timeline of the immune response to *Nippostrongylus brasiliensis* infection.

A systematic assessment of the immune response throughout *N. brasiliensis* infection highlights the lung as an essential location of immune orchestration (Figure 1). While the first encounter of the pathogen occurs at the epithelium when larvae penetrate the skin and enter circulation, this event is relatively quick (0–6 h). Nonetheless, there will be some local irritation and epithelial damage that recruits neutrophils and eosinophils to the tissue. The most extensive tissue damage occurs after the parasite has traveled through the vasculature and invades the lung parenchyma via alveolar capillaries in the airspace between 18 and 72 h after infection (31). This leads to hemorrhage and acute lung injury, partly mediated through neutrophil accumulation and activation (31). Epithelial cell-derived trefoil factor 2 controls helminth induced hemorrhagic lung injury and is necessary for IL-33 production (75). Resulting epithelial injury releases IL-25 and IL-33, both of which prompt type-2 cytokine production first from type-2 innate lymphoid cells (ILC2s) and, later, Th2 cells. After entry into the lung parenchyma, L3 larvae undergo maturation to the L4 stage and migrate to the airways where they travel up the pharynx and are swallowed. Upon entering the upper small intestine, L4 larvae latch onto the epithelium to feed, mature into L5 larvae, and produce eggs which are excreted in feces. Similar to the lung, damaged intestinal epithelium produces IL-25 and IL-33 further promoting the weep and sweep response. In mice with intact immune systems, the helminth is cleared within 8–10 days after infection. This is also when adaptive Th2 cells and eosinophils predominate as the major type-2 producing immune populations in the lung (56). Of note, the pathology observed in the lung after day 7 of infection by *N. brasiliensis* strongly resembles that of human asthmatic airways (32, 76).

As discussed above, tissue alarmins IL-25 and IL-33 are essential to ILC2 function in anti-helminth immunity. While damaged/dying epithelium represent important sources of these alarmins during the initial stages of helminth infection, many additional cellular sources have been described which likely impact ILC2 cells later in the response. For example, IL-33 is produced by adventitial stromal cells during helminth infection whereas type II pneumocytes and white adipose tissue-resident stromal stem cells produce IL-33 in other settings of type-2 inflammation (77–80). Similarly, IL-25 has been shown to be produced by many type-2 immune cells including Th2 cells, mast cells, alveolar macrophages, eosinophils and basophils—all of which may contribute to ILC2 activation (81–84). These cellular sources are in addition to more recently described IL-25-producing intestinal tuft cells and chemosensory brush cells found in the lung which play key roles in ILC2 homeostasis and expansion in settings of type-2 inflammation (85–89).

As initial responders of tissue damage caused by helminth infection, ILC2s serve a unique role in orchestrating the type-2 response that is required for worm clearance and epithelial repair. A detailed understanding of the immune response, especially that of ILC2s, is requisite to address the public health needs of treating helminth infections and preventing associated comorbidities.

## Group 2 Innate Lymphoid Cells

### ILC2s and Anti-helminth Immunity

#### Discovery of ILC2s

In 2001, a rare non-B/non-T (NBNT) cell population that responded to IL-25 and produced type-2 cytokines was described in settings of allergic inflammation (81). These cells resembled CD4^+^ T cells in many ways but lacked a known antigen receptor. It was proposed that this population worked in concert with Th2 cells to promote type-2 immune hallmarks. This IL-25-responsive NBNT population was later observed in the context of helminth infection where it was described as a prominent producer of type-2 cytokines during infection (90). However, it was not until almost a decade after their initial discovery that this NBNT population became part of the collective consciousness of researchers studying type-2 immunity (91–93). These studies showed that this innate lymphoid population rapidly produces IL-13 in response to the tissue alarmins IL-33 and IL-25. It was later shown that innate lymphoid cells could respond to the tissue alarmin TSLP to produce IL-5 and IL-13, but this appears to occur mainly in the skin leaving the impact of TSLP in other stages of helminth infection less clear (94, 95). While early studies used different names to describe innate lymphoid cells, consensus was reached in 2013 to identify them as group 2 innate lymphoid cells (ILC2s) based on their production of type-2 cytokines, distinguishing them from other innate lymphocytes classified as group 1 and group 3 ILCs (96). Over the last 10 years, significant advances have been made in elucidating the unique biological roles for all three subsets of ILCs, but ILC2s appear uniquely suited to respond to helminth infections and aid in the promotion of a protective type-2 immune response (97).

While wild-type mice can clear *N. brasiliensis* within 8–10 days of infection, mice lacking T cells and B cells remain colonized with worms after several weeks of infection (59). Although this highlights the essential nature of Th2 cells in productive worm clearance, additional importance of ILC2s in anti-helminth immunity is 2-fold. Not only do these cells contribute to parasite expulsion by orchestrating early cytokine production via their sensing of tissue alarmins, but ILC2s are also involved in mucosal barrier homeostasis and the reparative response after helminth-mediated tissue damage. The potential contribution of ILC2s in parasite clearance can first be seen in a study from 2006 prior to the discovery of ILC2s where IL-25-deficient mice which exhibited impaired worm clearance relative to wild-type mice, despite the presence of T cells (90). Looking back at this study, ILC2s can be directly implicated in this process as RAG-deficient mice (lacking B and T cells) given recombinant IL-25 cleared *N. brasiliensis* within 5 days of infection (90). These results were replicated in the first studies defining ILC2s where it was shown that IL-33 could also induce rapid helminth clearance, linking both IL-25 and IL-33 as early activators of ILC2s (92, 93). Indeed, transfer of ILC2s back into mice deficient in both T cells and ILCs (*Rag2*^−/−^*Il2r*γ^−/−^ mice) was sufficient to promote worm clearance (91, 93). It is important to note that this rapid clearance following alarmin administration was dependent on type-2 cytokines and particularly IL-13-expressing ILC2 cells (57, 90, 92, 93). In addition to their role in primary infection, ILC2 cells in concert with memory Th2 cells effectively limit worm burden and larval-induced lung damage upon secondary infection implicating a broader yet less defined role for ILC2 cells after repeated helminth exposure (98).

#### ILC2s in Barrier Homeostasis

In addition to their role in helminth expulsion, ILC2s in the small intestine serve an important function in barrier homeostasis via recognition of IL-25 made by chemosensory tuft cells residing in the epithelium (85–87). At steady state, dietary polysaccharides and metabolites bind directly to receptors on epithelial tuft cells and regulate their production of IL-25 (86, 99, 100). ILC2s in the intestinal lamina propria sense the tuft cell-derived IL-25. This maintains a low level of IL-13 production by intestinal resident ILC2s which then cues stem cells to differentiate into relatively low numbers of goblet and tuft cells (101). Upon colonization of the intestine by helminths such as *N. brasiliensis* and *H. polygyrus*, tuft cell expansion is increased (85–87, 102). Tuft cells act as early sentinels of intestinal infection by recognizing metabolites generated by pathogens that breech the mucosal barrier and produce substantially more IL-25 leading to increased ILC2-derived IL-13. This feed-forward circuit leads to the characteristic goblet and tuft cell hyperplasia observed in settings of type-2 inflammation. The increase in tuft cell-derived IL-25 as well as other alarmins generated as a result of tissue damage and cell death work in concert to promote type-2 immunity.

#### ILC2 Function

As discussed above, the lifecycle of the hookworm in the host causes a substantial amount of tissue damage and ILC2s provide an essential function in wound healing. This is particularly evident at two stages of infection in the lung: first when the infectious L3 larvae enter the lung parenchyma via the capillary endothelium, and then during the second stage of damage as L4 larvae migrate in search of the alveolar airspace. This is grossly observed in the lungs of mice 3 days after *N. brasiliensis* infection as punctate foci of damage and diffuse pulmonary hemorrhage. As sensors of epithelial damage, ILC2s are poised to respond quickly to such injury. It is clear that one of the major roles of tissue-resident ILC2s is to promote the mobilization of eosinophils in the bone marrow, which is enacted via their capacity to produce large amounts of IL-5 on a per cell basis (103). This corresponds to the characteristic eosinophilia that peaks around 9–12 days post-infection (73, 104). By day 12 in the response to *N. brasiliensis*, ILC2s are making IL-9 which is thought to autocrine amplify and be essential for tissue repair and restoration of lung function (105). Although IL-9 has a much lesser role in helminth immunity as compared to IL-4 and IL-13, ILC2-derived IL-9 likely enhances the production of IL-5 and IL-13, making this cytokine a potential modulator of the severity of type-2 inflammation (73, 74, 106). Also contributing to tissue repair is ILC2-derived epidermal growth factor (EGF)-like molecule amphiregulin (107). Though less is known about ILC2-generated amphiregulin in tissue repair within helminth infection, it is likely to be similar to that of other models with mucosal tissue damage. Such examples include the role of amphiregulin in modulation of dextran sulfate induced intestinal inflammation and influenza infection where ILC2-derived amphiregulin was critical in restoring barrier integrity and mediating airway remodeling (108, 109). A role for amphiregulin produced by ILC2 cells has also been observed in atopic dermatitis (110). In these models IL-33 induces the expression of amphiregulin in ILC2 cells, thereby promoting tissue repair. While the activity of ILC2-derived amphiregulin in tissue repair has not been studied in helminth models, it is likely to play a role as its expression is increased 12 days after *N. brasiliensis* infection (105). More recently, it was suggested that amphiregulin-producing ILC2s are a subset distinct from IL-5/IL-13-producing ILC2s (111). This study assessed pulmonary ILC2s at steady state in neonatal mice and used gene expression profiles to separate ILC2s into either *Klrg1/Il5/Il13*- or *Icos/amphiregulin*-expressing subsets. Future studies that identify and explore tissue-resident ILC2 heterogeneity may be able to assign differing roles for these subsets in anti-helminth immunity and other tissue-damaging infections or diseases.

### Inflammatory iILC2s and Anti-helminth Immunity

#### Phenotype

Until recently, ILC2s were largely considered to be a homogenous population. This was in contrast to ILC1s and ILC3s which consisted of distinct populations within each subset. However, evidence began to emerge that suggested heterogeneity within ILC2s. Early experiments using *Il1rl1*- (IL-33 receptor) and *Il17rb*- (IL-25 receptor) deficient mice revealed that IL-33 and IL-25 were not equal in their ability to promote type-2 hallmarks or helminth clearance (92). A theme became apparent in which IL-33 was the dominant alarmin associated with ILC2 activation, and IL-25 mediated a more selective role (112–114). Transcriptomics analyses later revealed distinct gene signatures in both mouse and human ILC2s further supporting that this innate lymphoid population was more diverse than previously believed (115–117).

Despite the suggested heterogeneity among ILC2s, the majority of studies to date were unable to assign distinct roles to different ILC2 subsets. The inability to reliably identify unique ILC2 subsets *in vivo* likely masked their separate but important contributions in anti-helminth immunity. However, this changed when a subset of ILC2s that could be distinguished by their differential responsiveness to an epithelial alarmin was characterized in the context of *N. brasiliensis* infection (118). This study identified a population of IL-25-responsive ILC2s that accumulated in the lungs of mice 5 days post-*N. brasiliensis* infection or in mice that were given intraperitoneal IL-25. These cells, which were coined inflammatory ILC2s or iILC2s, were not found in the lung at steady state and disappeared within 12 days after *N. brasiliensis* infection. This is in contrast to the ever-present, tissue-resident, IL-33-responsive ILC2 subset, termed natural ILC2s or nILC2s. This study also demonstrated that iILC2s phenotypically express high KLRG1 and low CD90 levels, whereas nILC2s have low KLRG1 but high CD90 expression. In some ways, iILC2s appear similar to IL-25-elicited MPP^type2^ cells as both populations are Lin^−^ c-Kit^+^ and respond to the same alarmin. However, MPP^type2^ cells are characterized by the lack of CD90 and IL-7Rα expression which distinguishes them from iILC2s (114, 118, 119).

**Figure 2:**
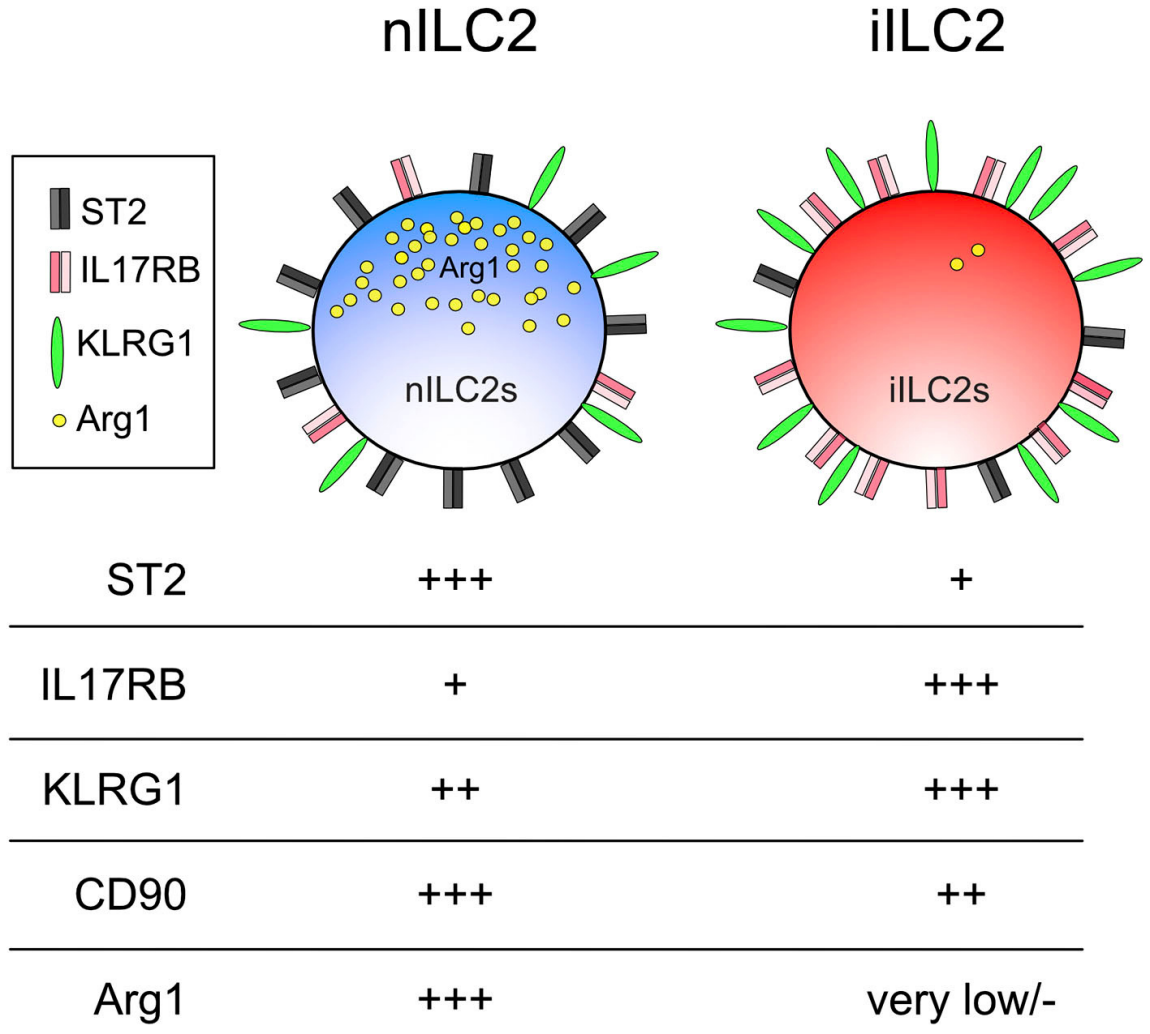
Phenotype of nILC2 vs. iILC2.

As their functional determinants imply, iILC2s which preferentially respond to IL-25 express more IL-25 receptor, whereas IL-33-responsive nILC2s display more IL-33 receptor on their surface (118, 120). Together, these markers allowed the consistent delineation between iILC2 and nILC2 cells (Figure 2). In addition to these cell surface markers, arginase 1 (Arg1) was later identified as a robust discriminator of the two subsets. It was previously thought that all ILC2s and their progenitors express this enzyme (121–123). However, while nILC2s display high Arg1 reporter expression using Arg1^YFP^ reporter mice, iILC2s express very little allowing for distinction between subsets (120). Interestingly, Arg1 expression in human pulmonary ILC2s obtained from individuals with chronic lung disease also appears to define two separate subsets. Human IL-33 receptor^+^ ILC2s displayed high Arg1 levels but not CRTH2^+^ ILC2s (123). Whether these two subsets in humans are related to nILC2s and iILC2s in mice is not yet known. Future investigations that delineate these ILC2 subsets using distinct phenotypic markers are likely to provide a more comprehensive understanding of the specific contributions allotted to these and potentially other ILC2 subsets.

#### iILC2 Function in Helminth Infection

The impact of iILC2 cells in anti-helminth immunity is demonstrated in mice lacking *Il25* or the IL-25 receptor *Il17rb* —which fail to specifically activate iILC2s—and display impaired *N. brasiliensis* expulsion (90, 92). This is complemented by experiments showing exogenous IL-25 administration is able to mediate *N. brasiliensis* clearance in mice lacking T cells but not in those lacking both T cells and ILCs (90, 93). Moreover, mice deficient for the AP-1 transcription factor BATF fail to generate pulmonary iILC2s and display impaired IL-25-mediated helminth clearance (40). Importantly, BATF deficiency had no impact on IL-33-mediated worm clearance or nILC2 numbers. Together, these data indicate that the IL-25-responsive iILC2 subset aids in mobilizing the immune response for rapid pathogen clearance and acts distinctly from tissue-resident IL-33-responsive subsets particularly in settings where IL-25 dominates the early alarmin response to infection.

It is logical that differences in cytokine production would exist between the ILC2 subsets. Tissue-resident pulmonary nILC2s, which are active at the peak of the response (days 8–10) to *N. brasiliensis*, are highly skewed toward IL-13 and IL-5 production with minimal IL-4 production (56, 57). Similarly, in response to intraperitoneal IL-25, there is a reported preference among iILC2s to produce IL-13 and little IL-4 as determined in 4C13R mice, which express AmCyan and DsRed-DR under *Il4* and *Il13* regulatory elements, respectively (118). However, in the context of *N. brasiliensis* infection, iILC2s make IL-4 in addition to IL-13 at 5 days post-infection (120). This study utilized the *IL4*^4*get*^, *IL4*^*KN*2^, *IL13*^*Yetcre*13^, and *IL13*^*Smart*13^ reporter mice which can be used as a readout of mRNA (*IL4*^4*get*^ and *IL13*^*Yetcre*13^) or protein (*IL4*^*KN*2^ and *IL13*^*Smart*13^) production (57, 93, 124, 125). Further differences between cytokine capabilities can be parsed out by assessing the temporal response to infection. At day 5 post-*N. brasiliensis* infection, cytokine production by ILC2s demonstrates that only the iILC2 compartment, and not nILC2s, are making type-2 cytokines (120). This is relatively early in the response and precedes cytokine production by tissue-resident IL-33-responsive nILC2s (126, 127).

While IL-13 is the major ILC2-derived cytokine in the intestine, IL-4 can promote goblet and tuft cell differentiation in organoid cultures similar to that observed after administration of recombinant IL-13, suggesting there may be a role for ILC2-derived IL-4 in maintaining intestinal barrier homeostasis (85–87, 128). Indeed, IL-4 production by intestinal ILC2s has been reported in response to *Heligmosomoides polygyrus* (129). Although this is an indication that ILC2-derived IL-4 in the intestine after *H. polygyrus* infection may play a role similar to that of early, migratory iILC2 cells in the lung after *N. brasiliensis* infection, some care should be noted. This study used IL-4-transcript reporter mice (*IL4*^4*get*^), which have been shown to mark ILC2 populations that are not readily producing IL-4 protein (56, 57, 93). Further complicating this conclusion, the prior study assessed cytokine protein only *ex vivo* after restimulation with phorbol 12-myristate 13-acetate (PMA) and ionomycin, a stimulus that drives translation of all cytokine-competent loci and may not reflect true *in vivo* cytokine production (56). Thus, it remains somewhat unclear if these intestinal ILC2 cells are actively producing IL-4 protein *in vivo*. If IL-4 protein production is confirmed, it is of interest to assess whether this IL-4 production reflects either: tissue-specific differences among nILC2 cells in the lung and intestine; helminth-specific differences related to infection by *N. brasiliensis* or *H. polygyrus*; or reflects an intestinal iILC2 population that maintains IL-4 protein production throughout the course of *H. polygyrus* infection.

Polyfunctionality and potential plasticity of iILC2s has also been described. Intraperitoneal IL-25 administration generated iILC2s that express elevated levels of Rorγt compared to nILC2s, though not as high as that of ILC3s from the small intestine (118). Furthermore, iILC2s were capable of producing IL-17 when stimulated with PMA and ionomycin, indicating potential plasticity between iILC2s and ILC3-like cells (118, 130). The polyfunctionality of iILC2s to produce IL-17 along with type-2 cytokines also sets them apart from nILC2s. This may indicate critical functional differences between the two subsets as iILC2s contribute to protection from the IL-17-sensitive pathogen *Candida albicans* (118). It may also reflect an inherent plasticity of iILC2 cells compared to nILC2 cells. Indeed, nILC2 cells require tissue specific signals in order to undergo their ultimate maturation (131). In support of ILC2/ILC3 plasticity, the lysine methyltransferase G9a could act as a switch to promote ILC3 and repress ILC2 commitment (132). Whether this epigenetic switch is active specifically in iILC2s or their progenitors relative to nILC2 cells is not clear. There is also some evidence to support ILC2/ILC3 plasticity in human ILC2s. Studies have shown two separate populations of human ILC2s delineated on c-Kit expression, as opposed to alarmin-receptor expression observed in mice. In these analyses, the c-Kit-positive subset exhibits similarities to ILC3s and is able to produce IL-17 (133, 134). The extent that ILC2 or ILC2 progenitors can regulate ILC3 responses will be an important area of future research as we try to better understand the importance of ILCs in barrier defense and repair. Future studies investigating the relative contribution of iILC2 compared to ILC3 populations would be of great interest toward defining their role in IL-17-mediated immunity.

#### Fate of iILC2s

The fate of iILC2s remains unclear. As iILC2s are only transiently found in the lung, it could be argued that they are relatively unimportant in the grand scheme of pulmonary immunity. However, the rapid disappearance of these cells has been attributed to their ability to convert to either the IL-33-responsive nILC2 population or IL-17-producing ILC3s (118). This study demonstrated that sorted iILC2s cultured under various conditions increased IL-33 receptor expression, a phenotype similar to that of nILC2s. *In vivo*, iILC2s transferred into congenic hosts upregulated the IL-33 receptor, again indicating a potential conversion to nILC2s (118). Furthermore, the fold increase of nILC2 cells after helminth infection was significantly reduced in *Il17rb*^−/−^ mice, which lack iILC2s, indicating a strong contribution of iILC2s to the nILC2 pool (118). However, studies using a tamoxifen-inducible fate mapping system that labels ILC2s concluded that repopulation and maintenance of the lung nILC2 pool after helminth infection was the result of self-renewal by tissue-resident nILC2s and not the addition of “*de novo*” generated ILC2s, which would include iILC2s (135, 136). Specifically, this system used an *Arg1*^*RFP*−*CreERT*2^ *R26R-YFP* mouse. In this mouse, all Arg1-expressing cells, including ILC2 precursors, were labeled with RFP and CreERT2. Administration of tamoxifen allowed Cre-mediated excision of the stop codon in the Rosa locus allowing permanent expression of YFP to fate map ILC2 populations throughout development. The majority of cells appeared to be self-renewed (i.e., retained YFP expression), which is consistent with prior literature suggesting self-renewal for both ILC1 and nILC2 populations (137, 138). Despite this, the fate mapping system identified that 12 days after a single *N. brasiliensis* infection, roughly 10–15% of nILC2s consisted of non-labeled cells (135, 136). This suggests that up to 15% of the tissue-resident pool may have been generated from converted iILC2s after barrier damage. Based on the knowledge that iILC2s are absent at 12 days post-*N. brasiliensis* infection, this time point is consistent with when iILC2s would have already converted to nILC2s. Thus, it is possible that although self-renewal is the predominant source of nILC2 homeostasis at rest, converting iILC2 cells may contribute substantially to the overall tissue-resident ILC2 pool over a lifetime of infections. Whether iILC2s are truly multipotent progenitors is not yet established, but these initial findings may indicate more plasticity among ILCs than previously appreciated as was recently reviewed (139).

Regarding experiments to assess conversion, it should be noted that while delineation of iILC2 and nILC2 populations based on KLRG1 expression works well for *N. brasiliensis* infection, there are other situations which lead nILC2s to increase KLRG1 levels. In particular, daily IL-33-administration for 4 days, as well as repopulation of irradiated mice with bone marrow, leads nILC2s to upregulate KLRG1 levels and drop CD90 slightly so that their phenotype resembles an intermediate between nILC2s and iILC2s (120). Moreover, these changes may be strain- and context-specific as *Alternaria alternata* exposure or IL-33 administration over 2 weeks lead to upregulation of CD90 on ILC2s isolated from bronchoalveolar lavage fluid in C57BL/6 but not BALB/c mice (140). In contrast, ILC2s isolated from lung tissue displayed downregulated CD90 only in BALB/c mice. Thus, using CD90 or KLRG1 expression levels to assess conversion may be confounded by inherent changes of these markers due to time, activation status, and tissue-specific signals. Further studies using more stringent markers of ILC2 subsets, such as Arg1, to determine conversion would strengthen the argument that iILC2s contribute substantially to the immune landscape of the lung.

#### Origin of iILC2s

**Figure 3:**
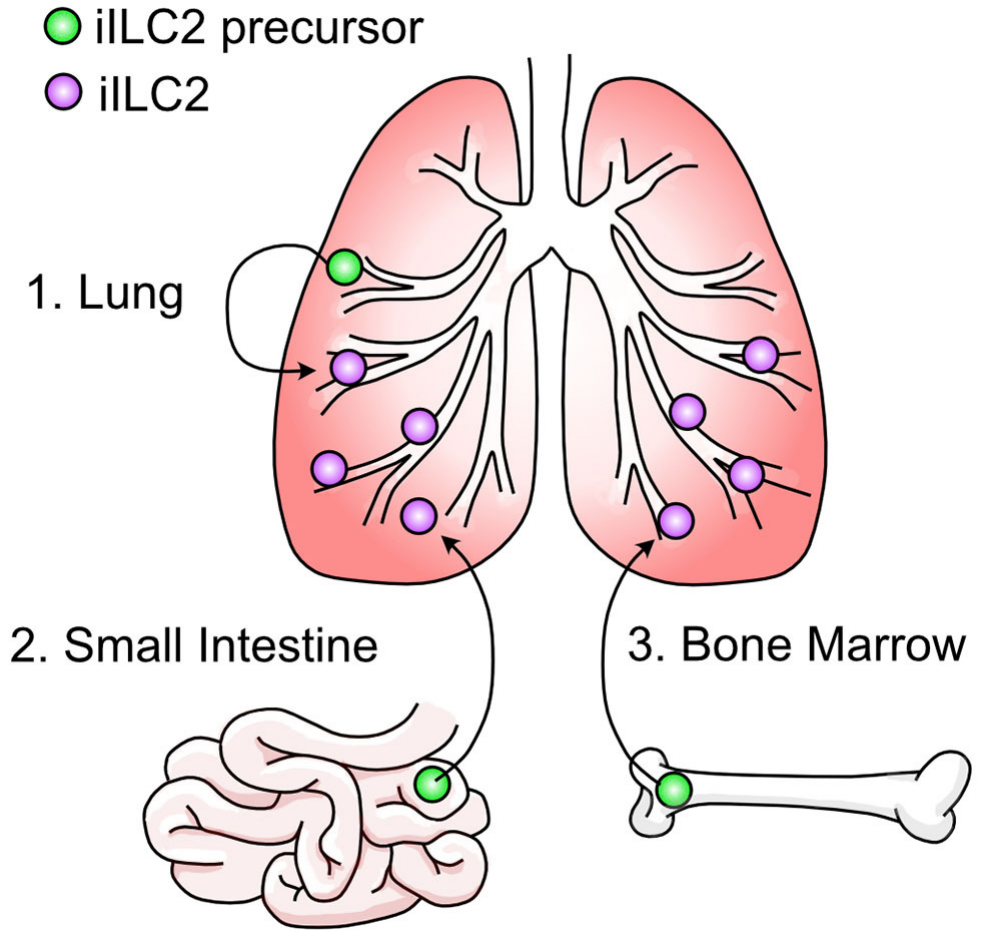
Three potential origins of pulmonary iILC2s.

It is not currently well understood where pulmonary iILC2s originate. As discussed, ILC2s have been considered tissue-resident cells that are replenished by self-renewal within their tissue of residence (131, 137). Using the fate mapping system described above where ILC2s are permanently labeled at various timepoints throughout development, it was demonstrated that the majority of lung-resident ILC2s in adulthood were derived during the postnatal period with some contribution of prenatal-derived cells and little influx of cells seeded during adulthood (135). This data explains the prior evidence that pulmonary type 2 immune cells—including ILC2s—arise in the lung shortly after birth (141–143). After *N. brasiliensis* infection, there was a statistically significant increase in non-fate mapped pulmonary ILC2s indicating that ILC2s do not arise solely from self-renewal during inflammation but may be recruited to the lung from other sites (135, 136). This is supported by studies where the appearance of iILC2s in the lung depends on S1P-dependent migration (120, 136, 144, 145). In these studies, administration of the S1P-receptor agonist FTY720 prevented the appearance of iILC2s in the lung after IL-25 administration or helminth infection, indicating the requirement for iILC2s to egress from lymphoid organs into circulation. Mucosal sites such as the small intestine and lung as well as sites of hematopoiesis like the bone marrow are all candidate reservoirs (Figure 3). Furthermore, evidence that c-Kit^+^ ILC precursors are also found in human blood, tonsils, and lung and can give rise to all ILC subsets indicates the potential for multiple sites of origin (146).

The most convincing source of migratory iILC2s in the lung has been the small intestine (136, 144, 145). In a comprehensive experiment to determine the source of iILC2s, total ILC2s were isolated from the lung, small intestine, and precursors from the bone marrow. When these cells were transferred into congenic hosts, pulmonary iILC2s arose in mice that received small intestine ILC2s (144). However, it should be noted that mice given ILC2 precursors from the bone marrow—but not the lung—also gave rise to pulmonary iILC2s, although to a lesser extent than those from intestine. It has been further demonstrated that IL-25-responsive iILC2s in the lung resemble small intestine ILC2s by transcriptional profile and phenotype (136, 144, 145, 147). Recently, a population of ILC2s resembling pulmonary iILC2s were found in mesenteric lymph nodes after *N. brasiliensis* infection which were absent in IL-33-deficient mice (147). Although migration of intestinal ILC2s to the mesenteric lymph nodes has been described previously (144), this study suggests that IL-33 binds to ST2^+^ nILC2s in the small intestine and through induction of the gene *Tph1*, generates ST2^−^ iILC2s. This mechanism would be consistent with the idea that pulmonary iILC2 cells originating in the intestine lose their nILC2 phenotype prior to their arrival in the lung. We previously proposed that such a conversion from an nILC2 phenotype toward an iILC2 phenotype must occur if pulmonary iILC2s originate in the intestine based on the uniform expression of the nILC2 marker arginase-1 within intestinal ILC2 cells and its absence among pulmonary iILC2 cells (120). However, because pulmonary and mesenteric iILC2 populations have not been directly compared, care should be taken in overinterpreting these conclusions at least as they relate to an intestinal origin for pulmonary iILC2 populations. In this regard, a few outstanding questions remain unresolved. First, addition of IL-25 and not IL-33 drives the appearance of pulmonary iILC2 cells suggesting that IL-33 may differentially impact mesenteric iILC2 and pulmonary iILC2 (118, 147). Second, while ST2 expression is abundant on nILC2 cells residing in the lung, few intestinal nILC2 cells express the IL-33 receptor at steady state (except in conditions where IL-33 expression in the intestine is forced by a transgene) (120, 147). How IL-33 is modulating intestinal ILC2s or whether specific ST2^+^ subsets are responsible for the generation of mesenteric iILC2s will be of interest. Third, while the mesenteric lymph node iILC2 population is observed 7 days post *N. brasiliensis* infection, pulmonary iILC2s are more transient and largely absent by this timepoint in the lung (118). Fourth, while iILC2s and nILC2s in the mesenteric lymph node or intestine express similar levels of KLRG1, iILC2s in the lung express noticeably more KLRG1 than pulmonary nILC2s. This discrepancy, along with that of arginase-1 expression as noted above, suggests that phenotypes of ILC2s likely differ depending on their tissue of residence making extrapolations based on phenotype with regard to cell origin difficult (118, 120, 136, 144, 145, 147). That said, there is evidence supporting that an intestinal origin for pulmonary iILC2 extends beyond studies with *N. brasiliensis*. Mice infected with the parasitic nematode *Trichinella spiralis*, which infects only the intestinal tract, demonstrated mobilization of iILC2s to the lung (145). These mice also displayed increased pulmonary mucin production due to iILC2-derived IL-13, indicating that an intestinal infection alone can mobilize iILC2s to the lung and drive mucosal immunity at distal sites. At present, conclusive demonstration of an intestinal origin for pulmonary iILC2s and its impact on iILC2 biology in the lung as well as investigation of whether other tissue sources outside the intestine contribute to pulmonary iILC2 cells await further experimentation.

In order for pulmonary iILC2s to originate from a lung source and undergo S1P-mediated migration, these cells would have to egress from the lung into circulation and then return back to the lung. This does not seem outside the realm of possibility, given the high degree of nILC2 motility and migration to perivascular spaces upon IL-33 treatment (148). In support, pulmonary ILC2s reside in adventitial niches allowing these cells the proximity to readily sample the vasculature (78). This scenario would also require iILC2s to be generated from nILC2s or a lung-resident precursor. Although *in vivo* support is limited, one study describes the requirement of Notch signaling for the generation of iILC2s, and when nILC2s were cultured on Notch ligand-expressing cells they adopted an iILC2 phenotype and gained the ability to produce IL-17 (130). Additionally, IL-18Rα^+^ ILC precursors have recently been reported in the murine lung that are capable of giving rise to nILC2s (111). It would be of interest to assess whether this lung tissue-resident precursor subset can also differentiate into iILC2s during helminth infection.

As the primary site of hematopoiesis in adults, the bone marrow also serves as a reservoir for ILC2 precursors (ILC2Ps). ILC2Ps have been defined as Lin^−^Sca1^hi^GATA3^hi^CD90^+^CD127^+^Id2^+^IL2rα^+^ (149). However, common ILC precursors and ILC2Ps in the bone marrow express gut homing molecules such as α4β7 and CCR9 making it difficult to acertain if such markers observed on lung iILC2 reflect an intestinal or bone marrow migrant (150). Further, even though lung ILC2s are seeded into their tissues of residence during pre- and postnatal development (135), there is evidence that IL-33 drives the egress of ILC2s from bone marrow progenitors (151). It is possible that other inflammatory signals may release iILC2 cells from a bone marrow progenitor, although this has not yet been assessed. Technical advances such as the generation of polychromic transcription factor reporter mice have been used to identify both uncommited ILC precursors and ILC2-specific precursors in the bone marrow as well as ILC2 precursors in the small intestine lamina propria (152). This would serve as an interesting model to identify the progenitor population as well as anatomical location that can give rise to iILC2s.

## Discussion

### Current Gaps in Knowledge and Future Directions

While the role of iILC2s in helminth infection is clear, there have been few studies demonstrating their appearance in other infection or disease models. As early mediators of type-2 immunity, it is interesting to speculate that iILC2s are involved in allergic responses. However, two recent studies have failed to demonstrate their recruitment to the lung using the house dust mite (HDM) model of allergic inflammation (120, 140). It is unknown whether IL-25 production is the main driver in iILC2 lung accumulation in the HDM setting or perhaps there is insufficient IL-25 production in the HDM model—and other models of type-2 immunity—to promote the expansion and migration of iILC2s to the lung. Disparities in pulmonary iILC2 accumulation between allergic and helminth models could also reflect differential involvement and location of IL-25-producing intestinal tuft cells and pulmonary brush cells. With this in mind, if iILC2s are first induced by intraperitoneal injections of IL-25 and then artificially transferred into T cell- and ILC2-deficient mice, they are able to mediate allergic responses upon HDM challenge (130). This data may indicate that while certain allergic responses do not normally recruit these cells to the lung, if iILC2 cells are already present in the lung (i.e., due to a helminth infection) at the time of allergen exposure, these cells may yet exacerbate type-2 inflammation. Whether such a mechanism explains why some individuals relocating to the United States from helminth-endemic regions show increased type-2 inflammation upon allergen exposure is not known (153). This is interesting to consider in context of mounting evidence that suggests helminth colonization suppresses the onset and severity of allergic disease (40, 154, 155). Although helminths are the focus here, other infections or insults that generate IL-25 and recruit iILC2s to the lung may similarly exacerbate allergic outcomes upon allergen exposure. Such models need to be explored to indicate iILC2 involvement.

While the use of reporter mouse systems have greatly impacted our overall understanding of ILC2 biology and provided unique opportunities to track the fate and function of these rare cells *in vivo*, it is necessary to acknowledge the inherent pitfalls associated with such systems. Reporter systems are often designed to enhance detection. This leads to alterations in gene regulation as well as mRNA and protein stability that may not reflect true biology of the wildtype gene. Aside from reporter “leakiness” where fluorescence or surface molecules may falsely indicate gene expression, there may also be contextual nuances that are missed. Gene expression and regulation is complex and sensitive to cell type, location, immune environment, and many other factors, each of which may lead to over-interpretation of results if not rigorously evaluated. As such, while innovative reporter models are likely to continue to be an important part of an investigators tool kit, complementation with unbiased approaches is becoming standard. An excellent example of this is the increasing use of single cell RNA sequencing to explore ILC2 biology. We expect platforms designed to interrogate the transcriptomics, genomics, proteomics, and metabolomics of ILC2 cells at the single cell level, when paired with traditional methods, will be critical to our collective understanding of ILC2s in settings of helminth infection.

Most importantly, how iILC2s contribute to the short- and long-term pulmonary landscape will be of great clinical interest. Eventually it will be necessary to detect pulmonary iILC2s in helminth-infected humans. If iILC2s convert to nILC2s and are retained in the lung, there would be significant contribution of these former iILC2s to the lung-resident population over the course of a lifetime as each infection could potentially contribute to the pulmonary ILC2 pool. In this scenario, *de novo* ILC2 populations like iILC2 cells, rather than self-renewing, tissue-resident populations, would likely become the predominant ILC2 population in mucosal sites. Such studies would expand our understanding of basic lung immunity, while continuing to inform the development of novel therapeutic strategies aimed at reducing the global health burden of STHs.

## Author Contributions

MM and RR wrote the review. Both authors contributed to the article and approved the submitted version.

### Conflict of Interest

The authors declare that the research was conducted in the absence of any commercial or financial relationships that could be construed as a potential conflict of interest.
